# Retraction: Downregulation of long non-coding RNA ANRIL suppresses lymphangiogenesis and lymphatic metastasis in colorectal cancer

**DOI:** 10.18632/oncotarget.28725

**Published:** 2025-05-09

**Authors:** Zhenqiang Sun, Chunlin Ou, Weiguo Ren, Xiang Xie, Xiayu Li, Guiyuan Li

**Affiliations:** ^1^Key Laboratory of Carcinogenesis of Ministry of Health and Key Laboratory of Carcinogenesis and Cancer Invasion of Ministry of Education, Cancer Research Institute, Central South University, Changsha, 410000, Hunan, China; ^2^Department of Gastrointestinal Surgery, Affiliated Tumor Hospital, Xinjiang Medical University, Urumqi, 830011, Xinjiang, China; ^3^Department of Gastroenterology, The Third Xiangya Hospital of Central South University, Changsha, 410000, Hunan, China; ^4^Hunan Key Laboratory of Nonresolving Inflammation and Cancer and Disease Genome Research Center, The Third Xiangya Hospital, Central South University, Changsha, 410000, Hunan, China; ^5^Hunan Provincial Tumor Hospital and the Affiliated Tumor Hospital of Xiangya Medical School, Central South University, Changsha, 410013, Hunan, China; ^6^Department of Coronary Artery Disease, Heart Center, First Affiliated Hospital of Xinjiang Medical University, Urumqi, 830011, Xinjiang, China

**This article has been retracted:** Oncotarget has concluded its investigation of this paper. Our concerns include internal duplication and overlaps with unrelated papers from different institutions. Specifically, we found that Figure 11, panels B and C, contain multiple duplicate tumor images and the b-actin image in Figure 4B overlaps with the b-actin panel in Figure 10. Moreover, Figure 6, illustrating invasion assay data, has duplicated panels found in two 2015 papers, in Figures 7, 11, and 12 of [1] and Figure 8 of [2]. In addition, all the panels of the tube formation assay in Figure 7 are duplicates of panels in Figure 3A of an earlier published, unrelated paper [3]. The migration assay images in Figure 8 also found in unrelated paper [4] and in Figures 5 and 6 from an already retracted article [5]. The Western blot images of VEGF-C in Figure 10 are duplicates of unrelated western blot images in earlier published papers [6, 7, 8] and all Western blot images in Figure 15A are duplicates from Figure 5F of [9].

Following Oncotarget’s initial inquiry regarding instances of image duplication, the first author, Zhenqiang Sun, acknowledged “two small errors in the paper” and wished to make corrections. However, the journal has since confirmed many additional instances of image duplication and notified all authors. We informed the authors that a retraction would be issued if they failed to respond to these concerns, but we received no reply. Therefore, the Editorial decision was made to retract this paper.

Original article: Oncotarget. 2016; 7:47536–47555. 47536-47555. https://doi.org/10.18632/oncotarget.9868

## References

[1] Li X , Chen L , Wang W , Meng FB , Zhao RT , Chen Y . MicroRNA-150 Inhibits Cell Invasion and Migration and Is Downregulated in Human Osteosarcoma. Cytogenet Genome Res. 2015; 146:124–35. 10.1159/000437379. . Retraction in: Cytogenet Genome Res. 2022; 162:95. DOI: 10.1159/000523671 PMID: 35231908 26361218

[2] Liu N , Jiang F , He TL , Zhang JK , Zhao J , Wang C , Jiang GX , Cao LP , Kang PC , Zhong XY , Lin TY , Cui YF . The Roles of MicroRNA-122 Overexpression in Inhibiting Proliferation and Invasion and Stimulating Apoptosis of Human Cholangiocarcinoma Cells. Sci Rep. 2015; 5:16566. 10.1038/srep16566. 26686459 PMC4685305

[3] Zhang L , Wang JH , Liang RX , Huang ST , Xu J , Yuan LJ , Huang L , Zhou Y , Yu XJ , Wu SY , Luo RZ , Yun JP , Jia WH , Zheng M . RASSF8 downregulation promotes lymphangiogenesis and metastasis in esophageal squamous cell carcinoma. Oncotarget. 2015; 6:34510–24. 10.18632/oncotarget.5923. 26439687 PMC4741469

[4] Xu D , Ma P , Gao G , Gui Y , Niu X , Jin B . MicroRNA-383 expression regulates proliferation, migration, invasion, and apoptosis in human glioma cells. Tumour Biol. 2015; 36:7743–53. 10.1007/s13277-015-3378-2. 25936342

[5] Li YJ , Dong M , Kong FM , Zhou JP , Liang D , Xue HZ . MicroRNA-371-5p targets SOX2 in gastric cancer. Oncotarget. 2016; 7:31993–2005. 10.18632/oncotarget.8289. . Retraction in: Oncotarget. 2018; 9:32551. DOI: 10.18632/oncotarget.26037 PMID: 30197762 27015119 PMC5077991

[6] Wang S , Wang X , Guo Q , Wang G , Han X , Li X , Shi ZW , He W . MicroRNA-126 Overexpression Inhibits Proliferation and Invasion in Osteosarcoma Cells. Technol Cancer Res Treat. 2016; 15:NP49–59. 10.1177/1533034615601563. 26319109

[7] Li JH , Luo N , Zhong MZ , Xiao ZQ , Wang JX , Yao XY , Peng Y , Cao J . Inhibition of microRNA-196a might reverse cisplatin resistance of A549/DDP non-small-cell lung cancer cell line. Tumour Biol. 2016; 37:2387–94. 10.1007/s13277-015-4017-7. 26376998

[8] He QY , Wang GC , Zhang H , Tong DK , Ding C , Liu K , Ji F , Zhu X , Yang S . miR-106a-5p Suppresses the Proliferation, Migration, and Invasion of Osteosarcoma Cells by Targeting HMGA2. DNA Cell Biol. 2016; 35:506–20. 10.1089/dna.2015.3121. . Retraction in: DNA Cell Biol. 2020; 39:1912. DOI: 10.1089/dna.2015.3121 PMID: 32955930 27383537

[9] Zhang L , Ding Y , Yuan Z , Liu J , Sun J , Lei F , Wu S , Li S , Zhang D . MicroRNA-500 sustains nuclear factor-κB activation and induces gastric cancer cell proliferation and resistance to apoptosis. Oncotarget. 2015; 6:2483–95. 10.18632/oncotarget.2800. 25595906 PMC4385865

